# Women in cardiology: critical status and a call to move forward

**DOI:** 10.1186/s43044-020-00078-w

**Published:** 2020-07-27

**Authors:** Hasan Ali Farhan, Zainab Atiyah Dakhil

**Affiliations:** 1 Scientific Council of Cardiology, Iraqi Board for Medical Specializations- Baghdad Heart Centre, Baghdad, Iraq; 2 Department of Medicine, Al-Kindy College of Medicine, University of Baghdad, Baghdad, Iraq

**Keywords:** Female, Fellowship, Gender gap, Developing countries, Middle East, Training

## Abstract

**Background:**

Healthcare workforce should mirror the population in representing patients’ diversity; however, in certain medical specialties like cardiology, there is a significant under-representation of females in fellowship programs. There is limited data discussing this issue in the Middle East, and up to our knowledge, no prior literature has cast a light on this subject in Iraq.

**Main text:**

Women represent not a minority but rather a negligible proportion of cardiologists in the Middle East, in general, and in Iraq, in particular, as over two decades, recruiting females in cardiology training never progressed. Women are facing many challenges that explain this gender gap, mainly work–life balance and risk of exposure to radiation in addition to society’s perceptions in the Middle East that underestimate women in interventional specialties.

**Conclusions:**

Serious efforts and forward steps should be taken by decision makers in cardiology fellowship programs and national cardiology societies to bridge this gender gap in order to improve cardiovascular care for both genders regardless of social barriers and traditional customs and to offer more access of care to those female patients who wish to be treated by female doctors based on their personal convictions.

## Main text

“**I raise up my voice-not so I can shout but so that those without a voice can be heard … we cannot succeed when half of us are held back**.” (Malala Yousafzai)

### Women in cardiology in emerging countries: where are they?

Gender gap in cardiology is not exclusive on female patients but penetrates substantially into female physicians. Cardiology nowadays is progressing at a rapid pace, making it a very attractive and competitive field with a wide spectrum of creative capabilities; however, in Iraq, women are certainly out of the picture as gender segregation in cardiology is outstanding compared with any other male-dominant specialties like surgery. Over the last decade, training in general surgery in Iraq witnessed a dramatic shift in the proportions of females attending the field, as the 2009 surgery fellowship program recruited 3 females among 35 trainees (8.6%) and the 2019 program recruited 33 females among 45 candidates (73.3%), while the cardiology program never experienced such a progress in this regard over the last two decades. Women in cardiology in Iraq are not a minority, but more to be a negligible proportion. The national cardiology fellowship program (3-year training program) was founded in 1994; since then, only three female adult cardiologists were recruited among 80 candidates. Moreover, since 2002, 53 fellows completed interventional cardiology diploma which is a 2-year training program; all of them were males. All these cardiologists are serving at 22 cardiac centers across the country (data from the Iraqi Scientific Council of Cardiology and Baghdad University/College of medicine database).

Taking in consideration that Iraqi population was estimated to be more than 38 million in 2018, with females constituting 49% (data from the Iraqi Ministry of Planning-Central Statistical Organization), this indicates a huge gender gap in representing population among healthcare professionals. Gender inequality in cardiology as being a male-dominated field is a perpetual rather than a historical dilemma.

**Fig. 1:**
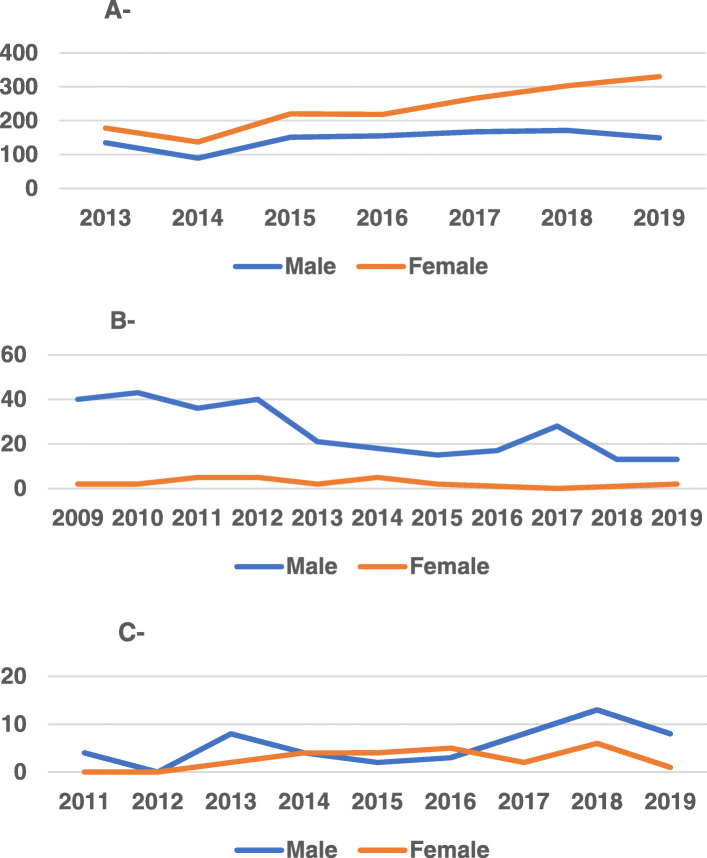
Gender distribution in undergraduate and postgraduate medical studies. **a** Gender distribution in graduates from medical colleges 2013–2019. **b** Gender distribution in internal medicine fellowship graduates 2009–2019. **c** Gender distribution in echocardiography diploma graduates 2011–2019. Females represent the higher proportion among medical college graduates, yet females constitute only minority of internal medicine fellows. Females represent better proportion in echocardiography diploma due to more predictable working hours and being a less stressful specialty (data from Baghdad and Al-Nahrain Universities and Iraqi Board for Medical Specializations database)

Females represent very large proportion of graduates from college of medicine, yet less to opt internal medicine and certainly cardiology as a career; internal medicine fellowship includes 4 years of training, and interventional cardiology needs additional 3 years of training; so the length of path and being a stressful specialty make women prefer other specialties like echocardiography diploma as it includes 2 years of training only (Fig. 1).

Despite different policies and cultural attitudes, other countries reported an under-representation of females in cardiology specialty, like in the USA where females constitute 21.5% of adult general cardiology fellows and 9.8% of interventional cardiology fellows in 2016 compared to 42.6% of internal medicine fellows [1]. From year 2006 to 2016, percentages of female adult cardiology fellows increased from 16.7 to 20.2%, and practicing adult cardiologists increased from 8.9 to 12.6%, yet both are considered low [1]. Taking the UK as another example where cardiology specialty has the highest number of consultants and among the highest number of fellows, females only constitute 13% of the consultants and 28% of the fellows which are among the lowest across medical specialties there [2]. In other European countries like Switzerland, women represent 26% of the cardiology staff from 2012 to 2017; the specialist title of cardiologist was obtained by 34% females versus 66% males, while in Belgium, 19.4% of cardiologists were women [3, 4]. Generally, ESC (European Society of Cardiology) reported that 25% of their members are women [4]. In emerging countries, these percentages are far lower. In Middle Eastern countries, the number of graduated males and females are almost near; however, the women who pursue cardiology and interventional cardiology are less than 5% [5]. It had been mentioned that the number of female cardiologists constitute 7% of the cardiology workforce in Oman [6]. In Lebanon, there are 18 female cardiologists (3% of all cardiologists) including only 2 interventional cardiologists, versus 477 male cardiologists [7]. Given all the above, we can say that gender gap in representing women in the cardiology workforce is a global issue. Nonetheless, it is more remarkable in Middle Eastern countries including Iraq.

Main hindrances preventing females from considering cardiology should be explored; some are related to their personal lives like work–life balance issues, radiation exposure that might conflict family planning and pregnancy, and time constraints in addition to dual work (unpaid work of childcare and households) making extra pressures on female doctors [8, 9]. Unlike males who are, especially in the Middle East, dominating and giving themselves the priority in both academic and professional practice achievements, unfortunately, this attitude is totally acceptable by the society and females themselves. Other factors are related to work environment, like gender discrimination in this field, which is not only from colleagues, but also from decision makers, allied healthcare professionals, and patients. Furthermore, female doctors have a deep belief that they will not advance in their career as expected mainly due to the inequity in training compared to their male peers, demanding work hours, and highly competitive work atmosphere.

Cardiology fellowship programs are of high standards; competency in many fields like echocardiography, Holter interpretation, coronary catheterization, device implantation, and programming is needed. In order to learn and upgrade such skills, extra hours of training are needed that mostly are out of working hours which makes cardiology specialty with unpredictable working schedule. Female doctors usually favor a specialty with less working hours and family friendliness when deciding their future career [10].

Another important issue that cannot be overlooked, being in a developing country, is the social prejudice on women in interventional specialties (other than gynecology) as they are usually underestimated and undervalued. Furthermore, there is consistent pressure from family members like husbands in pursuing a career with more contact with female patients like gynecology, pediatrics, or family medicine or a career with flexible working hours like dermatology or avoiding contact with patients as possible like in pathology or radiology. Moreover, there are interesting consistent suggestions to females from their male and female colleagues not to opt to cardiology as a career.

In hindsight, it seems obvious that the workforce should mirror the diverse patient population we treat. Although some decision makers may have grasped that insight, most cardiology community in developing countries have not, in such a way that gender issue in cardiology is still a minor concern to the cardiology society.

### How to bridge the gender gap?

Gender gap in cardiovascular medicine should be brought into view by stakeholders from all parties in meetings and conferences whenever possible. Practical steps should be undertaken to overcome the problem; the cardiology society should believe that the hurdle should be cleared instead of stumbling at it.

Across many surveys, women in cardiology usually have high career satisfaction level [11]. This should be invested because it means that it is difficult to recruit them but not so to maintain them in the field. It is important that the Council has the primary responsibility in initiating and enhancing females’ recruitment in cardiology by giving them the privilege when applied for fellowship program and giving them the option not to be exposed to radiation; and being non-invasive cardiologists, it is not only interventionists that we need; clinical cardiologists, academic cardiologists, cardiac imaging specialists, and programmers are all needed. More focus on the work atmosphere is needed by emphasizing on mentors, trainers, and decision makers to be supportive and value work–life balance of female physicians. Job sharing and flexible training hours (part-time working) can be suggested. There should be coordination with the internal medicine council to encourage junior doctors who show intention to consider a career in cardiology and embrace them morally and scientifically in order to limit the loss of creative talents and fellows with ambitious spirits from this field. Both the Scientific Council and national societies should intervene and address the radiation hazards that fellows and cardiologists are exposed to and should call for safety precautions and badge monitors to be obligatory as well as call for proper financial compensation for radiation hazards.

The national cardiology society should start taking the lead in supporting women, not only in cardiology but also in internal medicine specialty who may attend cardiology by encouragement into the field with research grants, travel grants, conferences and meetings attendance facilities. International meetings that discuss the role of females in cardiology are of paramount importance to be attended like ESC (European Society of Cardiology), EAPCI women (European Association of Percutaneous Cardiovascular Interventions) in cardiology meetings, ACC (American College of Cardiology), and CRT (Cardiovascular Research Technology). Hosting national female cardiologists in conferences and meetings will help to recruit more females into the field. Furthermore, international female cardiologists with pioneering works, trials, and achievements can be hosted in meetings and conferences in emerging countries, so that role modelling goal is achieved, which was reported by researchers to have an astonishing impact on female trainees, as female leaders in cardiology are the driving engine towards recruiting females in cardiology and retaining them [9, 10, 12]. Another vital yet overlooked aspect is national meeting and conference speakers as increasing female conveners, coordinators, panels, and speakers in national cardiology society meetings will increase gender balance at these meetings. In emerging countries, women are still represented rarely in leading positions both on academic levels like deans, editors in chief, and medical societies’ or council’s decision makers and on practice levels like hospital managers, department heads, or catheterization lab managers, so we need more women in powerful positions. Interestingly, we noticed that media has an influential impact on doctors in choosing their career, when popular medical TV shows started to glow like House and Grey’s Anatomy, many fellows were primarily affected by these series, and subsequently, they attended internal medicine or surgery as a career; this also can be invested, by making short movies or documentaries on pioneering female cardiologists and their achievements.

Overcoming the gender gap in cardiology is of paramount importance considering the social barriers and customs that are commonly encountered in the Middle East that prevent and sometimes forbid female patients from being examined by male doctors. Women should have confidence in themselves and should speak out and start the change, not wait for others to do so. More importantly, they should know the power of their voices, and we as decision makers and part of cardiology society should start paving the path for future females pursuing cardiology in order to make leading women in cardiology the tomorrow’s reality so let the voice be heard, stand up and step forward to bridge the gender gap.

## Data Availability

Not applicable

## Abbreviations

ACC: American College of Cardiology
CRT: Cardiovascular Research Technology
EAPCI: European Association of Percutaneous Cardiovascular Interventions
ESC: European Society of Cardiology
